# In Vitro Antioxidant, Anti-Inflammatory Activity and Bioaccessibility of Ethanolic Extracts from Mexican *Moringa oleifera* Leaf

**DOI:** 10.3390/foods13172709

**Published:** 2024-08-27

**Authors:** Erasmo Herman-Lara, Jesús Rodríguez-Miranda, Stefany Ávila-Manrique, Celia Dorado-López, Marisol Villalva, Laura Jaime, Susana Santoyo, Cecilia E. Martínez-Sánchez

**Affiliations:** 1 Tecnológico Nacional de México Campus, Tuxtepec, Calzada Victor Bravo Ahuja, No. 561, Col. Predio el Paraíso, San Juan Bautista Tuxtepec 68350, Oaxaca, Mexico; erasmo.hy@tuxtepec.tecnm.mx (E.H.-L.); jesus.rm@tuxtepec.tecnm.mx (J.R.-M.); 2 Institute of Food Science Research (CIAL), Universidad Autónoma de Madrid (CEI, UAM-CSIC), 28049 Madrid, Spain; stefyavilam@gmail.com (S.Á.-M.); cece.dorado@gmail.com (C.D.-L.); marisol.villalva@uam.es (M.V.); laura.jaime@uam.es (L.J.); susana.santoyo@uam.es (S.S.)

**Keywords:** anti-inflammatory, antioxidant, *Moringa oleifera*, pressurized fluids extraction

## Abstract

This study aimed to assess the antioxidant and anti-inflammatory properties, and bioaccessibility of *Moringa oleifera* ethanolic extracts using pressurized liquid extraction with varying ethanol concentrations (0%, 30%, 50%, 70%, and 100%) in water–ethanol mixtures. Quercetin derivatives and neochlorogenic acid were identified as major compounds via high-performance liquid chromatography with diode array detection. The 70% ethanol extract displayed the highest antioxidant activity and phenolic content, highlighting a strong correlation between phenolics and antioxidant potential. Extracts prepared with 50% and 70% ethanol (30 μg/mL) significantly inhibited TNF-α, IL-1β, and IL-6 cytokine secretion, with the 70% ethanol extract demonstrating robust anti-inflammatory effects. During in vitro digestion (oral, gastric, and intestinal phases), minimal changes were noted in most phenolic compounds’ post-oral phase, but reductions occurred after the gastric phase. Substantial decreases in major compounds and antioxidant activity were observed in post-gastric and intestinal phases. Overall, ethanolic extracts of *Moringa oleifera*, particularly those with 70% ethanol, exhibit promising antioxidant and anti-inflammatory properties, suggesting potential for developing therapeutic agents against oxidative stress and inflammation-related disorders. However, it is essential to protect these compounds to prevent their degradation during digestion.

## 1. Introduction

The Moringa tree (*Moringa oleifera*) is an important part of traditional Mexican horticulture. It is valued for its beauty and its uses in traditional medicine. Originally from the southern Himalayas—India, Bangladesh, Afghanistan, and Pakistan—it is now widely grown for tea, forage, and as a component in traditional medicine with other herbs [1,2]. In recent years, *M. oleifera* has received significant attention for being a rich source of bioactive compounds with diverse biological properties [3,4,5,6,7].

A large number of macronutrients, micronutrients, and secondary metabolites have been recognized in moringa leaves, such as carotenoids, polyphenols, phenolic acids, 10 flavonoids, alkaloids, glucosides, glucosinolates (thioglucoside), isothiocyanates, tannins, saponins, oxalates, and phytates [8,9]. The leaves are a rich source of omega 3, omega 6, and polyunsaturated fatty acids (represented by linoleic and linolenic acid). Palmitic acid occurs as the main saturated fatty acid. Regarding the mineral content, we can find potassium (K), calcium (Ca), and iron (Fe) and, to a lesser extent, magnesium (Mg) [10]. Among the vitamins, moringa leaves contain vitamin A, carotenoids, vitamin C, vitamin E (tocopherols), and some group B vitamins [9,11]. Dried moringa leaves are a source of polyphenols, such as phenolic acids and flavonoids. The main flavonoids that we find are myricetin, quercetin (also found as isoquercitin, that is, quercetin 3-*O*-glucoside), and kaempferol (also found as astragalin, that is, kaempferol 3-*O*-glucoside). However, the amount of these metabolites in *M. oleifera* extracts varies according to the geographical location, soil, sun exposure, and climatic conditions. Furthermore, the method and solvents used for its extraction can also modify the content of plant compounds, mainly phenols and flavonoids [12].

The modern food industry is now focused on developing functional foods and ingredients that aim to reduce disease risks [13]. This change reflects growing consumer interest in healthier dietary choices and the exploration of natural sources of functional ingredients [14]. At the same time, traditional medicine continues to use plants—whether fresh, dried, or in infusions and poultices—to treat a wide range of ailments. Despite these developments, research on Mexican *M. oleifera* using pressurized liquid extraction (PLE) to evaluate its antioxidant and anti-inflammatory activities in vitro is still limited. In vitro digestion models are widely used in research to predict the bioaccessibility of the studied compounds due to several advantages with respect to in vivo models, since they are relatively cheap and simple, faster, do not present ethical restrictions, the conditions can be controlled, sampling is easy, and the results are reproducible [15]. Furthermore, the evaluation of bioaccessibility using this type of model is well correlated with data obtained in animal and human studies [16]. Bioaccessibility has been defined as the amount of an ingested nutrient that is released from a food matrix and is available for intestinal absorption [17]. In the case of phenolic compounds, different food matrices and plant extracts have been subjected to gastrointestinal digestion in order to evaluate their bioaccessibility. Some studies have shown that phenolic compounds can modify their structure during digestion and, therefore, affect their biological activity. One of the most important factors to consider in this digestive process is the change in pH, since this can cause the decomposition and/or transformation of compounds [18]. An example is the case of the study carried out by [19] in which they determined that the carnosic acid present in a rosemary extract was transformed into carnosol due to a possible formation of the epoxy group due to the effect of intestinal pH.

Likewise, interactions with digestive enzymes, bile salts, or other compounds, in addition to other effects (precipitations, dimerizations, or conversions [20,21,22]) can also modify the chemical structure of phenolic compounds and, therefore, their bioaccessibility, thus affecting their biological activity [23].

Therefore, this study aims to evaluate ethanolic extracts from Mexican *M. oleifera* leaves, with a focus on their potential as natural sources of antioxidant and anti-inflammatory compounds. Additionally, this study aims to assess the in vitro bioaccessibility of the phenolic compounds extracted using pressurized fluids.

While *M. oleifera* has been studied extensively for its bioactive compounds, this study uniquely applies pressurized liquid extraction (PLE) with varying ethanol concentrations to optimize the extraction of phenolic compounds. This systematic approach to varying ethanol concentrations in PLE to assess both the yield and bioactivity of the extracts is innovative and adds depth to the existing body of knowledge on *M. oleifera*. Also, this study provides a thorough investigation of the antioxidant and anti-inflammatory properties of the extracts, specifically highlighting how different ethanol concentrations influence these properties. The in vitro digestion model used to evaluate the bioaccessibility of phenolic compounds adds a critical dimension to the research.

## 2. Materials and Methods

### 2.1. Preparation of Samples

*M. oleifera* leaves were harvested in San Juan Bautista Tuxtepec, Oaxaca, and dried in the shade at temperatures ranging from 27 to 30 °C over three days. After drying, the leaves were ground to achieve a particle size of 0.59 mm and subsequently sieved. Moisture content of the samples was 8.73% ± 0.12. The results for the extracts were reported on a dry weight basis. Analytical reagents and standards (reagent grade and HPLC) were purchased from Sigma-Aldrich, Spain.

### 2.2. Pressurized Liquid Extraction (PLE)

Extraction of *M. oleifera* samples was conducted using a Dionex system (Dionex Corporation, Sunnyvale, CA, USA) equipped with a solvent controller unit. Five ethanol: water (*v*/*v*) solutions with concentrations of 0:100, 30:70, 50:50, 70:30, and 100:0 were employed as extraction solvents. Each sample (1.0 g) was combined with 4.0 g of sea sand in a 10 mL stainless steel cell. The extraction cell was automatically filled with the appropriate solvent under a pressure of 1500 psi. The heating time for the extraction cell was 10 min at 100 °C. Following the heating phase, a static extraction was conducted. After the extraction, the cell was rinsed with a volume of solvent equivalent to 60% of the cell’s capacity. To ensure thorough removal of residual solvent, the cell was purged with pressurized nitrogen gas (N_2_) for 90 s. Additionally, a complete system rinse was performed between each extraction to prevent cross-contamination and ensure consistency. Following extraction, solvents were evaporated using an IKA RV 10 rotary evaporator (VWR International, Spain), and extracts were freeze-dried (Telstar Lyobeta 15 equipment; Telstar, Madrid, Spain). Powder extracts were kept at −20 °C until analysis [14].

### 2.3. Determination of Total Phenol Content (TPC) and Total Flavonoid Content (TFC)

TPC was assessed using the Folin–Ciocalteau method [24] with results expressed as mg gallic acid equivalents (GAE) per gram. A 125 mL sample was combined with 500 mL of distilled water and 125 mL of Folin–Ciocalteu reagent, and the mixture was allowed to stand undisturbed for 6 min. Following this, 1.25 mL of a 7% Na_2_CO_3_ solution and 1 mL of distilled water were added. The resulting mixture was kept in the dark at 25 °C for 90 min without stirring. After the incubation period, a spectrophotometer (Cary 60 UV-Vis, Agilent Technologies Inc., Santa Clara, CA, USA) was used to measure the absorbance at 760 nm. In parallel, a control procedure was conducted using distilled water. Each sample was analyzed in triplicate. Total flavonoid content (TFC) was determined using the method described by Heimler et al. [24]. In a test tube, 1250 μL of deionized water was mixed with 100 μL of the sample or standard (with deionized water serving as the blank) and 75 μL of 5% NaNO_2_ solution. The mixture was allowed to stand for 6 min, after which 150 μL of 10% AlCl_3_ solution was added and left to stand for another 5 min. Subsequently, 500 μL of 1 M NaOH was added, and the volume was adjusted to 2.5 mL with 425 μL of deionized water. After a 30 min incubation period, the absorbance of the samples was measured at 510 nm using a spectrophotometer (Agilent Technologies, Cary 60 UV–Vis, USA). The results were expressed as mg of catechin equivalents (mg EC) per gram.

### 2.4. Determination of Antioxidant Activity (ABTS•+ and DPPH•)

The ABTS•+ radical scavenging activity was determined using the method described by Re et al. [25]. First, 77.6 mg of ABTS•+ reagent was dissolved in 20 mL of distilled water to prepare a 7 mM aqueous solution. Then, 13.2 mg of potassium persulfate (2.45 mM) was added to the ABTS•+ solution in an amber bottle. The mixture was homogenized, covered with aluminum foil, and incubated at room temperature in complete darkness for 12 to 16 h, allowing the formation of the ABTS•+ radical. The ABTS•+ radical solution, stable for up to two days under these conditions, was then diluted with absolute ethanol until an initial absorbance of 0.7 ± 0.05 at 732 nm was achieved. To assess the radical scavenging activity, the ABTS•+ solution was mixed with the samples at various concentrations (10,000, 5000, 2500, 1000, 500, 250, 50, and 25 mg/L) in a 0.1:10 (*v*/*v*) ratio. The decrease in absorbance was measured, and the antiradical activity was calculated. The DPPH• (2,2-diphenyl-1-picrylhydrazyl) assay was performed to evaluate the antioxidant activity of the samples. First, 2.4 mg of DPPH• was dissolved in 100 mL of methanol to prepare the DPPH• solution. The samples were initially prepared at a concentration of 10,000 mg/L, followed by serial dilutions to obtain concentrations of 5000, 2500, 1000, and 500 mg/L. For the assay, 975 μL of the DPPH• solution was added to 25 μL of each sample in aluminum-lined Eppendorf tubes. The mixtures were incubated in total darkness for 15 min to ensure consistent reaction conditions. After the incubation period, the absorbance of each sample was measured at 515 nm using a spectrophotometer (Agilent Technologies, Cary 60 UV–Vis, USA). The antioxidant activity of the samples was quantified and expressed as mmol of Trolox equivalents per gram of extract (TEAC value) [25].

### 2.5. Analysis of Phenolic Compounds by HPLC-DAD

The phenolic compounds in the extracts were identified using high-performance liquid chromatography (HPLC). An Infinity 1260 chromatograph (Agilent Technologies, Madrid, Spain) equipped with a photodiode array detector (PAD) was employed. Separation was achieved on a reverse-phase C18 column (ACE Excel 3 Super C18, Symta, Madrid, Spain) with dimensions of 150 mm × 4.6 mm and a particle size of 3 μm. The column was protected by an ACE 3 C18-AR pre-column (7 mm × 13 mm) containing the same stationary phase. The column temperature was maintained at 30 °C, and the injection volume was set to 40 μL. The mobile phase consisted of two solvents: (A) 2% aqueous formic acid and (B) acetonitrile with 2% formic acid. The flow rate was kept constant at 1 mL/min. The gradient elution program was structured as follows: 0 min: 0% B, 1 min: 100% A, 6 min: 85% A, 21 min: 75% A, 26 min: 65% A, 36 min: 50% A, 43 min: 0% A, 48 min: 100% B, 50 min: 100% A, and 55 min: 100% A. Before injection, all samples were diluted in 970 μL of MilliQ ultrapure water, and 370 μL of the internal standard (ethyl gallate) at a concentration of 1 mg/mL was added. The samples were then filtered through a 0.45 μm PVDF filter. Quantification of the phenolic compounds was carried out using external calibration curves with appropriate analytical standards. Detection wavelengths were set according to the compound class: Hydroxybenzoic acids and flavan-3-ols: 280 nm, Hydroxycinnamic acids, and stilbenes: 320 nm and Flavonols: 360 nm.

### 2.6. Cell Culture

To investigate the anti-inflammatory activity of the extracts, we used the THP-1 human monocyte cell line ATCC TIB-202, a human myelomonocytic cell line displaying macrophage-like activity. The cells were cultured and expanded in complete RPMI 1640 medium, which contained 1% HEPES, 100 U/mL penicillin, 100 µg/mL streptomycin, 2 mM L-glutamine, and 10% fetal bovine serum (FBS) (Gibco, Spain). Cultures were maintained at a density of 2–9 × 10^5^ cells/mL in a 37 °C incubator with 5% CO_2_. Cells from up to 25 passages were used for all experiments. Monocyte differentiation into macrophages was induced by treating the cells with 100 ng/mL phorbol 12-myristate 13-acetate (PMA) (Sigma-Aldrich, Madrid, Spain) for 48 h, as described by Takashiba et al. [26].

### 2.7. Cytotoxicity Assays

The cytotoxicity of the samples was assessed using the MTT assay, which measures mitochondrial respiration based on the reduction of 3-(4,5-dimethylthiazol-2-yl)-2,5-diphenyltetrazolium bromide (MTT) (Sigma-Aldrich, Spain) by succinate dehydrogenase, an enzyme in the mitochondria. This reduction converts MTT into formazan, a colored compound, with the amount of formazan formed being proportional to the number of viable cells. THP-1 cells (5 × 10^5^ cells per well) were plated in a 24-well plate. After differentiation into macrophages, the cells were treated with the samples at concentrations of 30, 20, and 10 µg/mL and incubated for 24 h. Following treatment, 0.5 mL of a 0.5 mg/mL MTT solution was added, and the cells were incubated for an additional 3 h at 37 °C with 5% CO_2_. To solubilize the formazan, the cells were lysed with a 1:1 mixture of dimethyl sulfoxide (DMSO) (Panreac, Barcelona, Spain) and ethanol. The absorbance of the resulting solution was measured at 540 nm using a plate reader (Sunrise Remote, Tecan, Barcelona, Spain). The optical density of the formazan in the control wells (without sample) was considered as 100% viability, providing a baseline for comparison [27].

### 2.8. Anti-Inflammatory Activity

Macrophages underwent treatment with various non-cytotoxic sample concentrations and were then stimulated with 0.05 μg/mL bacterial lipopolysaccharide (LPS) (Sigma-Aldrich, Spain) to induce a non-specific inflammatory response. After an incubation period of 24 h at 37 °C with 5% CO_2_, samples of the supernatant were collected and stored at −20 °C for subsequent analysis. Macrophages treated with LPS alone were used as positive controls, while non-stimulated macrophages served as the negative controls. The quantification of cytokines released into the culture medium was carried out using a sandwich enzyme-linked immunosorbent assay (ELISA) kit (BD Biosciences, Almaraz, Cáceres, Extremadura, Spain) in accordance with the manufacturer’s instructions. Three pro-inflammatory cytokines—IL-6, IL-1β, and TNF-α—were measured. The ELISA was conducted in 96-well plates (Falcon, Corning, VWR, Aurora, OH, USA) that were pre-treated for primary antibody adhesion. The plates were incubated overnight at 4 °C, then washed and equilibrated to room temperature for one hour. Standards for each cytokine and 10 μL of cell supernatant samples, along with their respective controls, were added to the wells. The plate was incubated in the dark for two hours, washed, and then incubated with the secondary antibody and enzyme conjugate for one hour. After washing, an enzyme substrate solution was added, and the reaction was stopped with a stop solution after 30 min. Absorbance was measured at 450 nm with a reference wavelength of 570 nm using a plate reader (Sunrise Remote, Tecan, Männedorf, Zurich, Switzerland) [28].

### 2.9. In Vitro Gastrointestinal Digestion

For the in vitro digestions, a Titrino plus 877 titrator (Metrohm, Herisau, Switzerland) equipped with a double-jacketed conical vessel and magnetic stirrer was employed and maintained at 37 °C with a constant stream of water. This setup includes an electrode for pH control and a dispenser for titrant solution. The digestion of the moringa extract samples was followed by the method proposed by Soler-Rivas et al. [19] with some adjustments. Initially, a sample solution was prepared at a concentration of 20 mg/mL in a solvent mixture. Specifically, 110 mg of the extract was dissolved in 5.5 mL of a 70:30 ethanol: water mixture.

The oral phase commenced with the addition of 0.1 mL of a salivary solution containing 9.3 mg of human salivary α-amylase type XIII-A in 1 mM calcium chloride at a concentration of 50 U/mg. The mixture was stirred at 37 °C for 2 min. Subsequently, the gastric phase involved adding 25 mL of gastric solution, consisting of 127.3 mg of porcine pepsin from gastric mucosa (536 U/mg), dissolved in 25 mL of acidified water adjusted to pH 2 with 6 M hydrochloric acid. This gastric mixture was incubated under agitation in the dark at 37 °C for one hour. Following the gastric phase, 0.463 mL of a 325 mM calcium chloride solution and 1.389 mL of a 3.25 M sodium chloride solution (Sigma-Aldrich, Madrid, Spain) were added. The pH of the mixture was then adjusted to 7.5 using 0.1 M NaOH to initiate the intestinal digestion phase. This phase began with the addition of 115.7 mg of bile salts and 2.8 mL of a pancreatic solution comprising 9.3 mg of pancreatin (Sigma-Aldrich, Madrid, Spain) in 2.8 mL of 10 mM trizma-maleate buffer. The mixture was stirred in the dark for 2 h, with periodic adjustments of NaOH to maintain a constant pH. Upon completion of digestion, the samples were filtered through a 0.45 μm PVDF filter.

### 2.10. Statistical Analysis

Data were analyzed using STATGRAPHICS Centurion XVII.II software. One-way analysis of variance (ANOVA) followed by the least significant difference (LSD) test were applied for mean discrimination (*p* ≤ 0.05). All experiments were performed in triplicate.

## 3. Results

### 3.1. Yield Results of the Hydroalcoholic Extracts Obtained

The extraction yields were initially determined and are presented in Table 1. The results showed a decrease in yield with an increasing ethanol concentration compared to water. Interestingly, there were no significant differences in the extracts obtained with 0% and 30% ethanol, or with 50% and 70% ethanol. However, using 100% ethanol as a solvent resulted in a roughly 50% reduction in the extraction yield. These findings are attributed to the compounds found in moringa leaves, which are more soluble in water than in ethanol. 

This is likely due to its strong solvent properties that may not efficiently extract all components from the plant material.

### 3.2. Total Phenol Content (TPC) and Total Flavonoid Content (TFC)

An increasing ethanol concentration is correlated with a higher total phenolic content (TPC) and total flavonoid content (TFC) in the extracts (Table 2). Specifically, the extract using 70% ethanol showed the highest phenolic compound content at 111.30 mg GAE/g. However, there was no significant difference (*p* > 0.05) between the 100% ethanol and ethanol-free extracts, which exhibited lower values at 76.34 and 73.06 mg GAE/g, respectively. This indicates that TPC increased proportionally with the ethanol concentration, peaking at 70% ethanol. Conversely, TPC decreased when the ethanol concentration was raised to 100%, suggesting the reduced solubility of phenolic compounds compared to water–ethanol blends. These results imply that phenolic compounds from *M. oleifera* leaves are more soluble in water–ethanol mixtures than in either water or pure ethanol alone. This finding aligns with previous research; for instance, Wang et al. [29] optimized flavonoid extraction (such as quercetin and kaempferol) from Moringa leaves using pressurized liquid extraction (PLE) with water–ethanol mixtures containing 70% ethanol. Similarly, Rodríguez-Pérez et al. [3] reported the enhanced extraction of phenolic compounds from Moringa leaves using ultrasound-assisted extraction with water–ethanol mixtures containing 50% ethanol. These findings underscore the importance of the ethanol concentration in optimizing the extraction of bioactive compounds from *M. oleifera*, particularly emphasizing the advantages of ethanol–water blends over pure ethanol or water alone.

The maximum total flavonoid content was achieved with a 100% ethanol extraction. The observed increase in flavonoid extraction with the ethanol percentage supports findings from other studies on phenolic compounds and flavonoid extraction from *M. oleifera* leaves [30,31,32]. Hence, ethanol proves effective for extracting flavonoids, as suggested by Nobossé et al. [33].

### 3.3. Antioxidant Activities (ABTS•^+^ and DPPH•)

Both ABTS•^+^ and DPPH• assays demonstrated increased values with higher ethanol concentrations (Table 2). However, a decline in antioxidant activity was noted with 100% ethanol extraction. Peak values for both ABTS•+ (0.52 mmol TEAC/g) and DPPH• (0.46 mmol TEAC/g) were recorded with 70% ethanol extracts. These results correlated well with the phenolic compound content in the extracts. The 70% ethanol extract, which exhibited the highest antioxidant activity among the tested methods, also contained the greatest amount of total phenolic compounds. Rocchetti et al. [6] and Saucedo-Pompa et al. [34] have similarly attributed antioxidant activity in plant extracts to the phenolic compound content. Udechukwu et al. [35] emphasized the role of phenolic compounds like chlorogenic acid, quercetin, gallic acid, rutin, and kaempferol in the antioxidant activity of *M. oleifera* leaf extracts. These findings are consistent with studies by Hossain et al. [36], Ahmed et al. [37], and Oyeniran et al. [38] on moringa leaf extracts.

**Figure 1:**
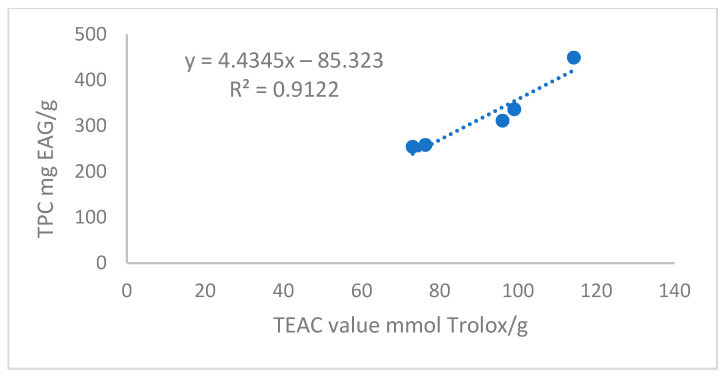
Correlation between TPC and Antioxidant Activity (TEAC Value) of the different extracts.

To establish a relationship between the TPC content and antioxidant activity, the DPPH• method was used to measure the antioxidant potential of the extracts. The findings (Table 2) reveal a peak in the antioxidant activity with 70% ethanol. Conversely, increasing the ethanol concentration to 100% markedly decreases the antioxidant activity. Consistent with these results, Braham et al. [31] demonstrated that extracts from moringa leaves using 50% water–ethanol mixtures and 70% ethanol exhibited higher levels of total phenolic compounds and greater antioxidant activity as assessed by the DPPH•method. When the total phenolic compound content of each extract is plotted against its antioxidant activity (Figure 1), a strong linear relationship between these variables emerges, with an R-squared value of 0.91. This result highlights a clear correlation between the antioxidant potential of moringa extracts and their phenolic compound content. This finding is in line with previous studies by authors such as Rocchetti et al. [6] and Saucedo-Pompa et al. [34], who have also linked the antioxidant activity in plant extracts to their phenolic compound concentrations. 

### 3.4. Phenolic Composition of the Extracts

**Figure 2:**
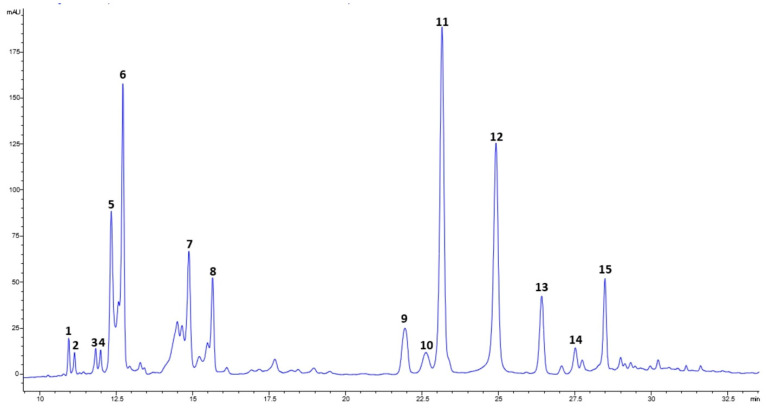
Chromatographic profile of moringa extract obtained with 70% ethanol.

The phenolic compounds present in the extracts were chemically characterized using HPLC-PAD. Table 3 shows the percentage composition of each component in the extracts. Among the prominent compounds were two derivatives of the flavonoid quercetin and neochlorogenic acid. One of the quercetin derivatives was identified as quercetin-*O*-hexoside, while the other remained unidentified. Generally, the more polar phenolic compounds (visible at the beginning of the chromatogram in Figure 2 were predominant in extracts using lower ethanol percentages (0 to 30%). Conversely, most phenolic compounds detected in moringa extracts showed increased extraction yields with higher ethanol percentages, peaking between 50% and 70%. Extracts prepared with 100% ethanol contained minimal amounts of polar phenolic compounds, focusing instead on fewer polar varieties. This finding is consistent with studies advocating ethanol or methanol–water blends for optimizing phenolic compound extraction from plant matrices [39,40,41]. The identified compounds (Table 3) align with previous reports on moringa leaf extracts [3,31,32,42,43].

### 3.5. Evaluation of Cytotoxicity and Anti-Inflammatory Activity of Moringa Extracts

#### 3.5.1. Cytotoxicity Evaluation

**Figure 3:**
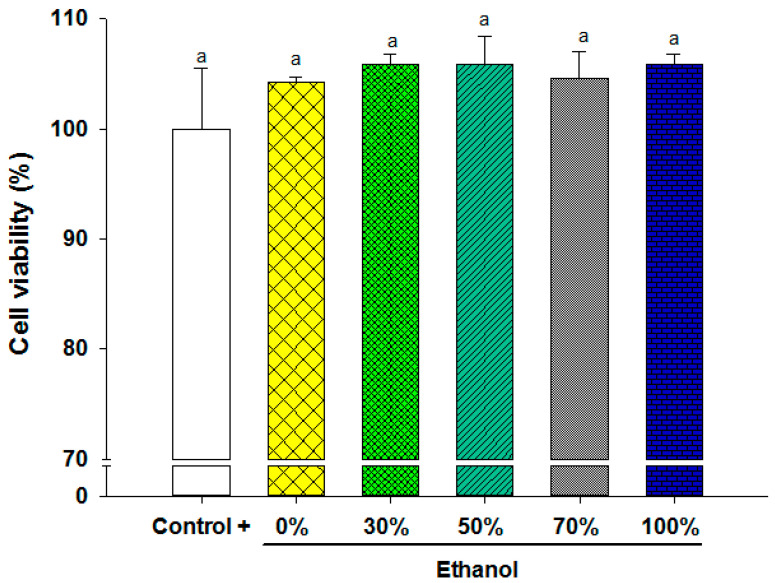
Cytotoxicity of the samples obtained with the different concentrations of ethanol in THP-1 cells differentiated to macrophages after 24 h, with a concentration of 30 µg/mL. Letter between columns indicate significant differences (*p* < 0.05).

Before evaluating the anti-inflammatory activity, cytotoxicity testing was conducted to determine non-toxic extract concentrations for cell cultures. Figure 3 illustrates the results for the highest concentration used (30 µg/mL), indicating no significant differences (*p* < 0.05) between extracts and controls across tested ethanol concentrations, thereby confirming non-cytotoxicity. According to the ISO 10993-5:2009 standard [40], if the relative cell viability for the highest concentration of the test sample is >70% of the control group, then the material shall be considered non-cytotoxic. Consequently, concentrations of 20 and 30 µg/mL were utilized in subsequent in vitro experiments.

#### 3.5.2. Anti-Inflammatory Activity

An anti-inflammatory activity assessment employed the THP-1 monocyte cell line, differentiated into macrophages by PMA induction to simulate inflammatory conditions. Macrophages were stimulated with LPS, with extracts at 20 and 30 µg/mL concentrations added post-cytotoxicity assessment. ELISA quantified pro-inflammatory cytokine secretion (TNF-α, IL-1β, IL-6) in the culture medium. Positive controls included LPS-stimulated macrophages without extracts, while negative controls represented unstimulated cells. 

**Figure 4:**
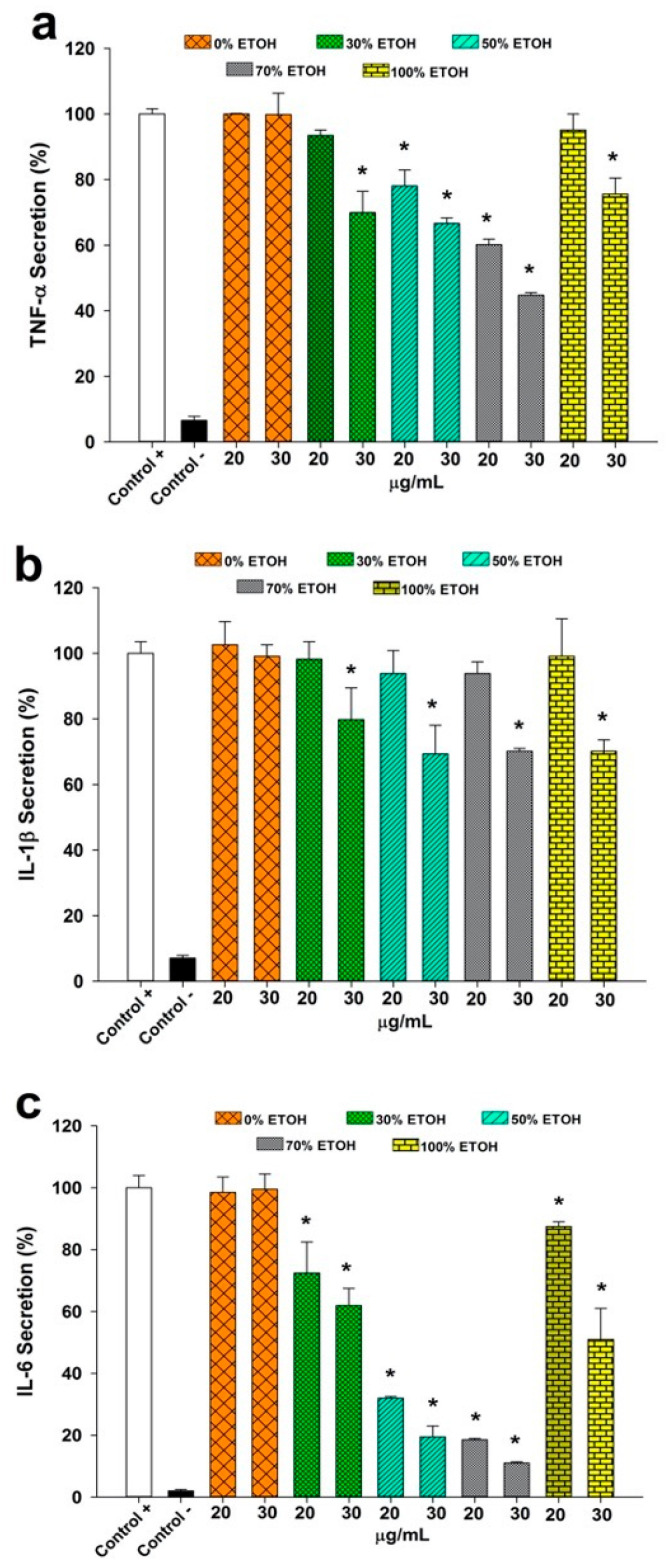
Percentage of secretion of (**a**) TNF-α, (**b**) IL-1β, and (**c**) IL-6 in macrophages after 24 h in the presence of the different extracted moringa extracts and different concentrations of ethanol–water, each with a concentration of 20 and 30 µg/mL. * Indicates the existence of significant differences with the control + (*p* < 0.05).

Figure 4a depicts the TNF-α results, showing that at 20 µg/mL, only extracts from 50% and 70% ethanol inhibited cytokine secretion relative to the positive controls, with the 70% ethanol extract demonstrating 40% greater inhibition compared to 50% ethanol. At 30 µg/mL, all extracts except water-based ones inhibited TNF-α release, with the 70% ethanol extract achieving the highest inhibition (60%). For IL-1β (Figure 4b), no inhibition was observed at 20 µg/mL; however, 30 µg/mL concentrations showed 30% inhibition with extracts from 50%, 70%, and 100% ethanol. The IL-6 results (Figure 4c) revealed no reduction in cytokine secretion for aqueous extracts, whereas those from 50% and 70% ethanol displayed potent (70%) inhibition at 20 µg/mL and 80% at 30 µg/mL.

Moringa extracts obtained with 50% and 70% ethanol demonstrated significant anti-inflammatory activity, effectively inhibiting the secretion of all three pro-inflammatory cytokines at 30 µg/mL concentrations. Notably, the 70% ethanol extract exhibited the highest activity, correlating with its elevated phenolic compound content. Further investigations using high-performance liquid chromatography-mass spectrometry (HPLC-MS) are recommended to delve deeper into the specific compounds responsible for this anti-inflammatory effect. Martínez-González et al. [4] also suggested that the in vivo anti-inflammatory activity of moringa leaf extracts could stem from compounds like chlorogenic acid and kaempferol-3-glucoside. Studies by Koheil et al. [44], Onsare et al. [45], and Prabakaran et al. [46] highlighted the anti-inflammatory, anti-rheumatic, anti-arthritic, and anti-cancer activities of ethanolic moringa leaf and seed extracts. These activities are attributed to phenolic compounds such as hydroxycinnamic acids, flavanols, hydroxybenzoic acids, and other phenolics [47]. Additionally, Ferreira et al. [48] identified primary antioxidant compounds like myricetin and quercetin, renowned for their diverse therapeutic applications. Zhang et al. [49] further demonstrated positive correlations between the polyphenol content and antioxidant and anti-inflammatory activities across 14 Chinese medicinal plants, gauging TNF-α secretion and NO production in murine macrophages.

### 3.6. Effect of In Vitro Digestion on the Phenolic Composition of Moringa Extract

**Figure 5:**
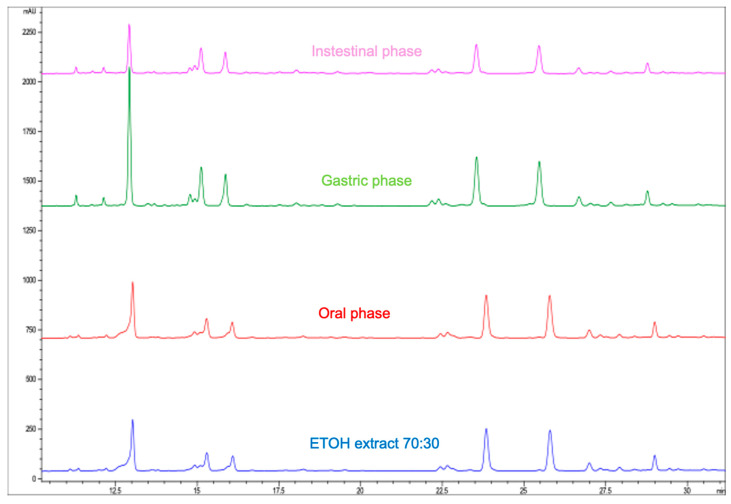
Shows the chromatographic profiles of the original extract after oral, gastric, and intestinal digestion phases.

The moringa extract obtained through Pressurized Liquid Extraction (PLE) using 70% ethanol is rich in phenolic compounds and has significant antioxidant properties. For these beneficial phenolic compounds to effectively unleash their potential in a living organism, they must be bioavailable, meaning they must successfully traverse the digestive system and be absorbed in the intestine. To investigate this, the moringa extract was subjected to an in vitro digestion simulation to assess how the process, including digestive enzymes and pH variations at different stages, could impact the extract’s phenolic compounds and antioxidant activity. The extraction’s in vitro digestion procedure followed the methodology outlined in Section 2. This protocol had been refined by the research group before. In addition, a control digestion was carried out, substituting the extract with a 70% ethanol–water mixture. Samples were collected after the oral, gastric, and intestinal phases in each digestion cycle to analyze how each phase of digestion affects the phenolic compounds of the extract. Figure 5 displays the chromatographic profiles of the original extract and after oral, gastric, and intestinal digestion phases. These results do not show differences between the profiles of the original extract and those obtained after the oral phase of digestion. After the gastric and intestinal phases, there are notable differences between the obtained profiles.

Table 4 illustrates the identification and quantification of various phenolic compounds after the different phases of digestion. Relative to the original extract, most of the quantified phenolic compounds show no significant differences after the oral phase. After the gastric phase, an increase in neochlorogenic acid is notable compared to its amount in the original extract. However, only minimal differences are observed for the other phenolic compounds after the gastric phase.

In this context, it can be stated that the gastric enzymes and acidic pH during this phase are unlikely to have a significant impact on most of the identified phenolic compounds, except for neochlorogenic acid. The observed increase in neochlorogenic acid may be attributed to transformations or degradation processes experienced by other compounds within the same family. However, after the intestinal phase, a reduction in most of the studied phenolic compounds is observed. This reduction is likely due to the action of intestinal enzymes and/or the basic pH, which can affect almost all the phenolic compounds to varying extents.

Most phenolic compounds, including quercetin-*O*-hexoside and its derivative, experience losses of approximately 17–20% after the intestinal phase. However, it is worth noting that neochlorogenic acid shows better stability, with around 69% of this compound being retained after the intestinal phase. The varying chemical structures of these compounds result in different sensitivities to changes in pH and the actions of digestive enzymes during the in vitro gastrointestinal digestion process. This observation has been highlighted by several authors [50,51]. In the majority of phenolic compounds, both quercetin-*O*-hexoside and its derivative experience losses of about 17–20% after the intestinal phase. However, it is important to note that neochlorogenic acid shows a higher percentage of recovery, retaining about 69% of the compound after the intestinal phase. This difference in stability during the in vitro gastrointestinal digestion process can be attributed to the distinct chemical structures of these compounds. 

Guo et al. [52] studied the stability of various phenolic compounds during simulated digestion. They found that compounds with specific structural characteristics, such as hydroxyl groups and glycosylation, were more stable compared to others. In a separate study, Reboul et al. [53] examined the digestion of phenolic compounds in fruits and vegetables, noting that stability varied depending on the specific compound and the food matrix. Significant variations in the release and bioaccessibility of phenolic compounds during digestion were observed. Ribeiro et al. [54] focused on the stability of phenolic compounds in beverages during simulated digestion and noted that compounds with ester bonds and higher molecular weights were more susceptible to degradation. Serra et al. [55] investigated the impact of digestion on the stability of phenolic compounds in olive oil and observed significant changes in the composition and bioavailability of these compounds during the digestion process. These studies emphasize the importance of considering the chemical structure of phenolic compounds when assessing their stability during gastrointestinal digestion.

#### Influence of In Vitro Digestion on Total Phenolic Compounds and Antioxidant Activity of Moringa Extract

This study also investigated how digestion affects the total phenolic compounds and antioxidant activity of the extract at each phase, as depicted in Table 5. The results show that the amount of total phenolic compounds in the moringa extract remains relatively unchanged after the oral and gastric phases compared to the original extract. However, there is a decrease in the quantity of total phenolic compounds after the intestinal phase. These findings are consistent with the HPLC-PAD analysis, which also demonstrates a reduction in phenolic compounds following the intestinal phase.

In terms of the sample’s antioxidant activity, it remained stable during the oral phase but experienced a significant decrease following the gastric and intestinal phases. Particularly noteworthy is the low antioxidant activity observed after the gastric phase, which is not consistent with the abundance of total phenolic compounds in the extract. This discrepancy might be attributed to a potential interaction between phenolic compounds and gastric enzymes, rendering them undetectable by HPLC analysis. Similar phenomena have been documented by other researchers [56], especially in the case of luteolin-7-*O*-glucoside. The sample’s antioxidant activity remained stable during the oral phase but showed a significant decrease after the gastric and intestinal phases. It is worth noting that the antioxidant activity was particularly low after the gastric phase, despite the high abundance of total phenolic compounds in the extract. This inconsistency may be due to a potential interaction between phenolic compounds and gastric enzymes, which makes them undetectable by HPLC analysis. 

## 4. Conclusions

This study on *M. oleifera* ethanolic extracts reveals several practical applications due to their potent antioxidant and anti-inflammatory properties. The identification of key compounds, particularly in 70% ethanol extracts, underscores their potential in nutraceuticals and functional foods aimed at managing oxidative stress and inflammation, both of which are linked to chronic diseases like cardiovascular conditions, diabetes, and cancer. This research provides a solid scientific foundation for incorporating *M. oleifera* into health-promoting products, reinforcing its role in preventive healthcare. This study’s findings on the strong anti-inflammatory effects of these extracts suggest that they could serve as natural sources for developing plant-based anti-inflammatory drugs or supplements, which are increasingly favored due to their lower risk of side-effects compared to synthetic alternatives. Moreover, the antioxidant properties of Moringa extracts present opportunities in the cosmetics industry, particularly in products designed to protect the skin from oxidative damage and aging. These extracts could be integrated into skincare formulations to help mitigate the effects of environmental stressors on the skin. Additionally, the potential use of Moringa extracts as natural preservatives in food products could extend the shelf life by preventing oxidation and spoilage, offering a natural alternative to synthetic preservatives. This study also explored the stability of these bioactive compounds during in vitro digestion, a critical factor for their efficacy when consumed. Understanding how these compounds behave throughout digestion can inform the development of delivery systems that protect and enhance their bioavailability, ensuring that consumers gain maximum health benefits from *M. oleifera* products.

## Figures and Tables

**Table 1:** Extraction yield of various extracts in dry basis.

Ethanol (%)	Yield (mg Extract/g)
0	33.73 ± 1.65 ^a^
30	33.89 ± 0.40 ^a^
50	31.34 ± 0.33 ^b^
70	29.27 ± 0.62 ^b^
100	16.00 ± 1.33 ^c^

Different superscript letters denote significant differences between samples in the same column (*p* < 0.05).

**Table 2:** Analysis of total phenolic compounds (TPC), total flavonoids (TFC), and antioxidant activity (ABTS•+ and DPPH•) in the various extracts.

Ethanol (%)	TPC (mg GAE/g Extract)	TFC (mg Q/g Extract)	ABTS•+ (mmol Trolox/g Extract)	DPPH• (mmol Trolox/g Extract)
0	73.06 ± 5.80 ^c^	18.80 ± 1.16 ^d^	0.36 ± 0.02 ^c^	0.26 ± 0.01 ^d^
30	96.04 ± 7.58 ^b^	24.90 ± 0.71 ^c^	0.41 ± 0.01 ^b^	0.31 ± 0.01 ^c^
50	99.07 ± 5.33 ^b^	29.85 ± 1.36 ^b^	0.43 ± 0.00 ^b^	0.34 ± 0.01 ^b^
70	111.30 ± 4.43 ^a^	33.20 ± 2.05 ^b^	0.52 ± 0.01 ^a^	0.46 ± 0.00 ^a^
100	76.34 ± 3.94 ^c^	51.00 ± 4.79 ^a^	0.27 ± 0.02 ^d^	0.26 ± 0.00 ^d^

Different letters in the same column indicate significant differences (*p* < 0.05) between the extracts.

**Table 3:** Phenolic composition of the different *Moringa oleifera* extracts.

Peak	Compound	Ethanol (%)				
		0	30	50	70	100
1	Unidentified	--	--	95.5 ± 0.3	90.9 ± 5.8	16.3 ± 0.6
2	Unidentified	125.4 ± 0.1	152.7 ± 0.3	69.8 ± 0.3	60.5 ± 2.9	--
3	Unidentified	--	--	70.7 ± 0.7	71.6 ± 1.7	--
4	Caffeoylquinic acid	124.7 ± 0.1	136.6 ± 2.5	68.9 ± 0.5	61.2 ± 2.1	--
5	Cryptochlorogenic acid derivative	26.9 ± 0.5	21.3 ± 0.4	408.5 ± 3.7	498.7 ± 34.4	502.7 ± 109.5
6	Neochlorogenic acid	1230.5 ± 0.8	1856.1 ± 2.3	1007.7 ± 1.8	875.3 ± 49.6	483.5 ± 30.9
7	Cryptochlorogenic acid	592.7 ± 0.2	797.6 ± 1.8	546.7 ± 0.4	497.2 ± 43.5	276.7 ± 8.2
8	Vicenina	560.5 ± 0.2	635.0 ± 1.1	694.8 ± 0.7	379.2 ± 16.5	200.7 ± 13.3
9	Vitexin	254.9 ± 0.6	383.7 ± 0.4	415.2 ± 0.4	406.3 ± 25.4	208.1 ± 14.8
10	Vitexin derivative	119.4 ± 0.5	124.8 ± 0.4	135.3 ± 0.1	169.7 ± 8.2	220.8 ± 17.1
11	Quercetin-O-hexoside	1314.3 ± 10.8	2093.2 ± 12.9	2308.6 ± 22.9	2627.5 ± 32.9	1657.8 ± 48.8
12	Quercetin derivative	1972.8 ± 0.7	2976.4 ± 4.6	3162.7 ± 3.4	2533.0 ± 20.7	348.2 ± 20.1
13	Kaempferol derivative	274.8 ± 0.1	424.6 ± 0.5	470.3 ± 0.1	524.8 ± 39.1	422.1 ± 13.0
14	Isorhamnetin-3-O-glucoside	60.7 ± 0.6	52.0 ± 0.6	72.8 ± 0.2	157.6 ± 3.3	186.3 ± 4.9
15	Kaempferol derivative	388.7 ± 0.2	583.8 ± 0.9	641.7 ± 0.1	543.3 ± 25.7	220.3 ± 6.9

Data expressed as area under the curve, RT: Retention time.

**Table 4:** Phenolic composition of the extract before and after oral, gastric, and intestinal digestion phases.

Phenolic Compounds	Content (mg/g Extract)			
	Extract	Oral	Gastric	Intestinal
Caffeoylquinic acid	0.28± 0.03 ^b^	0.33 ± 0.03 ^b^	0.52 ± 0.01 ^a^	0.47 ± 0.01 ^a^
Neochlorogenic acid	7.69 ± 0.02 ^b^	8.29 ± 0.18 ^ab^	9.58± 0.13 ^a^	5.28 ± 0.66 ^c^
Chlorogenic acid	3.10 ± 0.02 ^b^	3.46 ± 0.15 ^ab^	3.93 ± 0.11 ^a^	3.68 ± 0.47 ^a^
Vicenine	2.88 ± 0.38 ^c^	2.88 ± 0.03 ^c^	4.47 ± 0.26 ^a^	3.80 ± 0.01 ^b^
Luteolin glycoside	0.09 ± 0.02 ^a^	0.11 ± 0.02 ^a^	0.13 ± 0.02 ^a^	0.13 ± 0.03 ^a^
Luteolin glycoside derivative	0.02 ± 0.01 ^b^	0.03 ± 0.01 ^ab^	0.05 ± 0.01 ^a^	0.01 ± 0.01 ^b^
Vitexin isomer	0.54 ± 0.04 ^b^	0.58 ± 0.01 ^b^	0.69 ± 0.08 ^a^	0.53 ± 0.03 ^b^
Vitexin	0.73 ± 0.04 ^a^	0.77 ± 0.01 ^a^	0.72 ± 0.22 ^a^	0.65 ± 0.04 ^b^
Quercetin-*O*-hexoxide	13.87 ± 0.47 ^a^	14.48 ± 0.14 ^a^	13.44 ±0.79 ^a^	10.92 ± 0.60 ^b^
Isoramnetin-*O*-hexoxide	0.25 ± 0.09 ^b^	0.21 ± 0.30 ^b^	0.52 ± 0.26 ^a^	0.32 ± 0.16 ^b^
Quercetin derivative	14.54 ± 0.68 ^a^	14.52 ± 0.18 ^a^	12.97 ± 0.97 ^b^	12.12 ± 1.58 ^b^
Kaempferol derivative	2.89 ± 0.14 ^a^	3.01 ± 0.01 ^a^	2.93 ± 0.15 ^a^	2.47 ± 0.15 ^b^
Isorhammetin-*O*-hexoside	0.72 ± 0.01 ^b^	0.82 ± 0.00 ^a^	0.58 ± 0.10 ^c^	0.34 ± 0.09 ^d^
Quercetin derivative	0.52 ± 0.05 ^a^	0.56 ± 0.01 ^a^	0.53 ± 0.03 ^a^	0.42 ± 0.05 ^b^
Isorhammetin-*O*-hexoside	0.88 ± 0.05 ^a^	0.90 ± 0.06 ^a^	0.84 ±0.05 ^a^	0.74 ± 0.06 ^b^
Kaempferol-*O*- hexoside	4.35 ± 0.18 ^a^	4.65 ± 0.11 ^a^	3.51 ±0.61 ^b^	3.85 ± 0.29 ^b^
Quercetin derivative	0.33 ± 0.02 ^a^	0.36 ± 0.03 ^a^	0.30 ±0.02 ^a^	0.25 ± 0.05 ^b^
Kaempferol derivative	0.37 ± 0.03 ^a^	0.39 ± 0.04 ^a^	0.33 ±0.04 ^b^	0.38 ± 0.02 ^a^

Different superscript letters denote significant differences between the compounds after the different phases of digestion (*p* < 0.05).

**Table 5:** Total phenolic compounds and antioxidant activity of the extract during different phases of gastrointestinal digestion.

Samples	TPC (mg GAE/g)	DPPH• (mmol Trolox/g)
Moringa extract	76.21 ± 2.37 ^a^	0.308 ± 0.009 ^a^
Oral phase	78.81 ± 2.75 ^a^	0.314 ± 0.018 ^a^
Gastric phase	78.87 ± 2.17 ^a^	0.246 ± 0.008 ^c^
Intestinal phase	73.23 ± 1.98 ^b^	0.283 ± 0.019 ^b^

Different superscript letters denote significant differences between samples in the same column (*p* < 0.05).

## Data Availability

The data presented in this study are available on request from the corresponding author.
